# The Role of Augmented Reality in the Advancement of Minimally Invasive Surgery Procedures: A Scoping Review

**DOI:** 10.3390/bioengineering10040501

**Published:** 2023-04-21

**Authors:** Phillipp Brockmeyer, Bernhard Wiechens, Henning Schliephake

**Affiliations:** 1 Department of Oral and Maxillofacial Surgery, University Medical Center Goettingen, D-37075 Goettingen, Germany; 2 Department of Orthodontics, University Medical Center Goettingen, D-37075 Goettingen, Germany

**Keywords:** augmented reality (AR), virtual reality (VR), mixed reality (MR), minimal invasive surgery (MIS)

## Abstract

The purpose of this review was to analyze the evidence on the role of augmented reality (AR) in the improvement of minimally invasive surgical (MIS) procedures. A scoping literature search of the PubMed and ScienceDirect databases was performed to identify articles published in the last five years that addressed the direct impact of AR technology on MIS procedures or that addressed an area of education or clinical care that could potentially be used for MIS development. A total of 359 studies were screened and 31 articles were reviewed in depth and categorized into three main groups: Navigation, education and training, and user-environment interfaces. A comparison of studies within the different application groups showed that AR technology can be useful in various disciplines to advance the development of MIS. Although AR-guided navigation systems do not yet offer a precision advantage, benefits include improved ergonomics and visualization, as well as reduced surgical time and blood loss. Benefits can also be seen in improved education and training conditions and improved user-environment interfaces that can indirectly influence MIS procedures. However, there are still technical challenges that need to be addressed to demonstrate added value to patient care and should be evaluated in clinical trials with sufficient patient numbers or even in systematic reviews or meta-analyses.

## 1. Introduction

### 1.1. Advantages and Disadvantages of Minimally Invasive Surgery (MIS)

Advances in surgery have the potential to significantly improve patient outcomes and reduce hospital length of stay (LOS) [1]. Healthcare providers can achieve cost savings and improve efficiency in the delivery of healthcare services, and increase the capacity to treat more patients, thereby improving access to care and reducing waiting times [2]. To achieve these goals, it has been emphasized to continue to invest in surgical advances and technologies, such as minimally invasive surgery (MIS) techniques [3] and the development of new, innovative surgical tools [4].

The overall definition of MIS includes surgical procedures that reduce the morbidity of conventional surgical trauma [5]. These techniques have been made possible primarily by the development and adoption of endoscopic systems and the continued miniaturization of imaging systems [5]. Insufflation devices can be used for the controlled inflation of body cavities and the creation of surgical workspaces [5]. In the last decade, the development of robotic-assisted surgery has provided another technological advancement for MIS, which is currently gaining importance [6]. The main advantages of MIS reported in the literature include reduced postoperative pain due to the avoidance of extensive surgical trauma [5]. This may also reduce the number of immobility related morbidities such as postoperative atelectasis or venous thrombosis [7]. In addition, MIS can help to improve the visibility of inaccessible areas, reduce the risk of inflammation, and improve recovery time and cosmesis [5]. Consequently, they can significantly reduce the mean LOS of patients [8,9,10].

One of the major disadvantages of MIS is the extended surgical “learning curve” [11]. with complications occurring early during this period [11]. Watson et al. identified the first 20 procedures as the interval with the highest risk of complications [12]. This finding has led to the widespread use of educational courses, simulators, web-based videos, and mentoring programs in surgical education and training [5]. In addition, there are procedure-specific risks such as insufflation complications, port site metastasis after laparoscopic ablative surgery, and hernia formation and bleeding [5]. Dissections through robotic-assisted minimally invasive procedures generally take longer than traditional open surgical approaches [6], which may be associated with lower operative turnover rates and corresponding economic disadvantages [5]. However, this disadvantage is usually offset by lower costs associated with a shorter LOS [5]. Today, there is a great demand for MIS procedures, and there are some potential opportunities to develop them further.

### 1.2. The Basics of Augmented Reality (AR) and Its Impact on Healthcare

The development of new technologies has come to the forefront, especially during the Corona pandemic [13]. Virtual reality (VR) and augmented reality (AR), also referred to as mixed reality (MR) [14], are among the three-dimensional (3D) technologies that have been used in consumer marketing for many decades and are currently gaining momentum in healthcare [15].

AR is a technology that overlays computer-generated images or videos onto real-world objects and environments [16]. The use of AR has the potential to revolutionize the way surgery is delivered and received [17]. AR has been shown to improve patient outcomes [18], increase the efficiency and accuracy of surgical procedures [19], and enhance medical education and training [20]. AR technologies such as AR glasses [21], AR head-mounted displays [22], and AR smartphones [23] are being developed and deployed in healthcare settings, with some early applications showing promising results [23]. However, AR in healthcare is still in its early stages of development, and there are several technical and regulatory challenges that must be addressed before it can be widely adopted [24].

## 2. Materials and Methods

To address the question of the role of AR in improving MIS procedures, a scoping literature search was conducted in the PubMed and ScienceDirect databases to identify all relevant studies and reviews available from January 2018 to February 2023. Different areas of application were identified and the potential advantages and disadvantages of AR were evaluated.

This review article was designed and conducted according to the enhancing transparency in reporting the synthesis of qualitative research (ENTREQ) guidelines [25] to reveal and discuss current evidence on the utility of AR in MIS.

**Figure 1:**
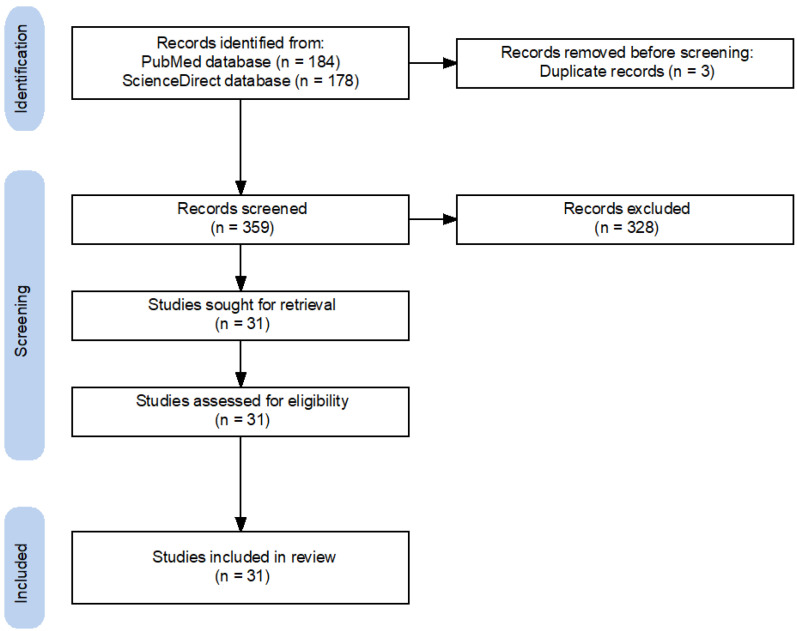
Preferred reporting items for systematic reviews and meta-analyses (PRISMA) flowchart of search strategy [27].

The search strategy followed the identification and screening guidelines by the preferred reporting items for systematic reviews and meta-analyses (PRISMA) statements [26]. The following Mesh search headings and keywords were used: “Augmented Reality”, “AR”, “surgery”, “minimal invasive surgery”, and “MIS”. These terms were used in different boolean combinations. We retrieved all eligible studies and evaluated the reference lists of the identified studies and reviews (Figure 1).

Articles were screened for relevance by two authors (PB and BW) based on title and abstract. In case of uncertainty, full-text screening was performed. A total of 31 articles were independently reviewed by both authors. All articles with questionable relevance were reviewed independently by both authors to reach a consensus. In case of disagreement, a third author (HS) was involved.

Articles were included if they were considered relevant to the advancement of MIS through AR technologies if they were relevant to the practice of MIS (i.e., procedures that fall within the scope of MIS), or its subspecialties if the method used to study AR could be applied to MIS (i.e., use of an AR headset or novel features) and if they had potential educational or didactic value for MIS.

Articles were excluded if they were not directly relevant to MIS techniques, did not address AR, or were not published in English. A total of 31 studies relevant to both AR and MIS promotion were identified. Articles were accessed through the University of Goettingen library system.

Due to the small number of randomized clinical trials, a risk of bias analysis was not required and was not performed.

## 3. Results

A total of 31 articles from the last five years (2018–2023) were included in the review. The role of AR in the improvement of MIS procedures was identified in three main areas: AR-guided navigation, improving education and training conditions, and building improved user-environment interfaces (Table 1). Some articles were found to be related to more than one primary theme.

### 3.1. AR-Guided Navigation

Of the 31 papers included a total of 16 articles addressed the topic of AR-based navigation. Six studies were experimental or proof-of-concept approaches. One study was a prospective multicenter clinical approach, one study was a retrospective analysis of clinical data, one study was a prospective case-controlled study, one study was a case report, one study was a clinical investigation, and five studies were review articles. Of these, two articles were systematic reviews. Existing studies and their reported outcome data showed a high degree of heterogeneity (Table 1), which inhibited a comprehensive comparison based on quantitative data.

In an experimental approach, Zadeh et al. analyzed the impact of artificial intelligence (AI) concepts using the Uteraug system, an AR-based guidance application for laparoscopic uterine surgery [28]. A dataset of 3800 images from 79 laparoscopic videos was created, including various annotations: uterine segmentation, uterine contours, and left and right tubal junction regions. The dataset was divided into a training and a test dataset, and a neural network was trained for automatic surgical image interpretation. The performance of the neural network on the training dataset was compared with that of experts on the test dataset. A performance plateau was demonstrated at 700 images for uterine segmentation and 2000 images for uterine contouring. The segmentation results for the training and test datasets were 94.6% and 84.9% (the higher the better), and the final contouring error was 19.5% and 47.3% (the lower the better). The authors concluded that this system could automatically interpret surgical images and provide AR performance equivalent to the current manual system by providing additional image datasets [28].

In their review, Xu et al. analyzed current surgical tracking systems, one of the most important technologies for MIS navigation, and evaluated the advantages and disadvantages of different solutions [29]. The problem of information loss is an inherent drawback of monomodal surgical navigation systems. It is characterized by physical limitations, attenuation, signal dropouts, and unstable accuracy of tracking algorithms. The authors conclude that future research trends, such as the integration of AR for visualization during surgical tracking, will tend to ameliorate this problem [29].

Butler and colleagues described the first in vivo use of AR-guided percutaneous MIS placement of pedicle screws in the setting of spine surgery [30]. A total of 164 MIS cases were performed on patients from June 2020 to March 2022 by three senior surgeons at two institutions. AR was used for percutaneous pedicle screw instrument placement with spinal navigation. A total of 606 pedicle screws were placed. The AR system used consisted of a wireless headset with a transparent near-eye display that projected intraoperative 3D images directly onto the surgeon’s retina. The intraoperative CT data were processed in the headset and integrated into the surgeon’s field of view, creating a “see-through” 3D effect in addition to the standard 2D navigation images. MIS pedicle screw placement was performed percutaneously through a single line of sight with navigated instruments. The average time from registration to percutaneous access to final screw placement was 3 min and 54 s per screw. Learning curve analysis showed that operative times were similar in early cases compared to those performed with more experience with the system. The authors noted the efficiency and safety of AR-guided screw placement compared to conventional technologies [30].

Zhu et al. proposed an AR-based neuroendoscopic navigation system to assist surgeons in the localization and removal of intracerebral hematomas [31]. Dual-mode AR navigation was proposed to provide comprehensive guidance from catheter implantation to hematoma removal. The authors developed a series of experiments to validate the accuracy and validity of this system. The average mean square error of registration between medical images and patients was 0.784 mm, and the variance was 0.1426 mm. The degree of pixel mismatch was less than 1% for the different AR modes. For the catheter implantation experiments, the mean distance error was 1.28 mm and the variance was 0.43 mm, while the mean angular error was 1.34° and the variance was 0.45°. Comparison experiments were performed to evaluate the practicality of the system. The authors concluded that this AR guidance system can provide stereo images with depth information fused to the patient to help surgeons locate targets and remove hematomas [31].

Lecointre and colleagues developed and tested an AR-based robotic assistance system that performed a real-time multimodal and temporal fusion of laparoscopic images with preoperative medical images in a porcine model [32]. It enabled targeted in vivo lymph node detection during minimally invasive pelvic lymphadenectomy. A measurement campaign was performed to determine the most accurate tracking system (UR5 cobot versus NDI Polaris). Subsequent procedures on two pigs consisted of identifying artificial target lymph nodes and anatomical landmarks without and with AR support. The AR overlay on the target structures was quantitatively evaluated. Clinical relevance was assessed by a questionnaire completed by experienced and trainee surgeons. The accuracy of the CT overlay was greater than 90% and the overflow rate was less than 6%. The authors describe the system as reliable, safe, and accurate, and plan to validate its use in a clinical trial [32].

Guo et al. evaluated the feasibility, accuracy, and efficacy of AR technology and 3D plate library assisted posterior MIS for scapular fractures [33]. They retrospectively evaluated the records of 21 patients with scapular fractures treated with posterior MIS with reconstruction plates: 9 patients were treated with conventional fixation, while 12 patients were treated with preoperative virtual simulation and intraoperative navigation-assisted fixation with the AR system. Operative time, blood loss, complications, and Hardegger function were compared between the two groups. It was found that the patients who used the AR system had significantly shorter operation time (−28.75 min; *p* = 0.0007) and lower blood loss (−81.94 mL, *p* = 0.0052) than the patients in the conventional surgery group. The authors concluded that AR and 3D plate library-assisted posterior MIS is an effective and reliable method for the treatment of scapular fractures, allowing precise preoperative planning and intraoperative navigation. It also saves time and allows for a more individualized treatment plan, resulting in a safer reduction procedure [33].

Felix et al. placed a total of 124 thoracolumbar pedicle screws under AR guidance in seven cadavers [34]. Sixty-five screws were placed in four donors using open spine surgery. Fifty-nine screws were placed in three donors using an MIS approach. AR was used exclusively for pedicle screw navigation in both open and minimally invasive spine surgery. A total of 124 pedicle screws were placed using AR navigation with 96% accuracy (Gertzbein-Robbins Class A and B). The combined angular error was 2.4° and the distance error was 1.9 mm. The authors concluded that AR is a highly accurate, emerging technology for navigation in open and minimally invasive spine surgery using commercially available headset hardware [34].

In their review, Yuk et al. present recent advances and innovations in the use of simulation methods in spine surgery [35]. These include VR, MR, and AR. While VR and MR are primarily used for teaching and surgical preparation, the authors describe an advantage of AR technology primarily in practical neurosurgical spine surgery situations [35].

Chen and colleagues proposed a novel in situ AR navigation system with enhanced arthroscopic information for knee surgery [36]. In an experimental approach, intraoperative anatomical positions were first determined using arthroscopic images and arthroscopic calibration, and then a tissue property-based model deformation method was proposed to update the preoperative 3D knee model with anatomical position information. The updated model was then rendered using a glasses-free 3D display to obtain a global AR view of the surgical field. In addition, virtual arthroscopic images were generated from the updated preoperative model to provide anatomical information about the surgical site. The experimental results showed that the virtual arthroscopic images could reproduce the correct structural information with a mean error of 0.32 mm. Compared to 2D arthroscopic navigation, AR navigation reduced target errors by 2.10 mm in the knee phantom and by 2.70 mm in the in vitro porcine knee experiment. The authors concluded that AR navigation is useful for minimally invasive knee surgery because it can provide global in situ information and detailed anatomical information [36].

In their systematic review, Benmahdjoub et al. examined AR technology and its utility in craniomaxillofacial surgery [37]. From a total of 7067 articles identified by AR and surgical keywords, 39 articles were selected. Based on these articles, a classification of study types, surgery types, devices used, metrics reported, and benefits was performed. The results suggest that AR technology could provide a number of benefits by overcoming the challenges of traditional navigation systems, such as hand-eye coordination and depth perception. However, the authors also point out that it is difficult to summarize the metrics reported in the articles, to obtain statistical values from the current studies, and that user evaluation studies are not yet available [37].

In their review, Hussain and colleagues evaluate the benefits and challenges of AR systems in skull base surgery [38]. The authors suggest that navigation systems incorporating AR offer comparable results to conventional navigation systems in terms of precision but with improved ergonomics and visualization. However, more needs to be conducted to improve the current state, achieve maximum safety and reliability, and reduce system costs [38].

Hussain and colleagues reviewed relevant features of AR navigation in MIS and examined its evolution over time [39]. They discussed key features relevant to surgical advancement, including technique and technology development, accuracy, overall healthcare costs, operating room time savings, and radiation exposure. They conclude that AR technology will make surgery safer and more efficient, and that fluoroscopy may be completely replaced by image guidance [39].

Hu et al. evaluated the clinical application of the augmented reality computer-assisted spine surgery (ARCASS) system for percutaneous vertebroplasty (PVP) in their prospective case-control study [40]. A total of 18 patients undergoing PVP with the ARCASS system were included. The control group consisted of age- and sex-matched patients who underwent standard PVP and met the same selection criteria as the case group. Compared to the control group, the ARCASS group required significantly fewer fluoroscopies (6 vs. 18, *p* < 0.001) and had a shorter operative time (78 vs. 205 s, *p* < 0.001) during entry point identification and local anesthesia, which began with the registration of the skin entry point at the lesion site and ended with identification of the bony entry point. In terms of accuracy, the ARCASS group had a significantly higher percentage of “good” entry points in the lateral view (81.8% vs. 30.0%, *p* = 0.028) and in the anteroposterior view (72.7% vs. 20.0%, *p* = 0.020) than the control group. The authors concluded that the AR system is clinically feasible for PVP and provides a more precise bone entry point with reduced operative time and unnecessary radiation exposure [40].

Gribaudo et al. presented an approach for developing real-time AR solutions for navigation in robotic surgery [41]. A modular approach was developed to solve the tracking problem in in vivo robotic surgery. The authors point out that by dividing the entire surgical procedure into a series of phases, it is possible to assign the best tracking strategy to each phase and to reuse implemented software mechanisms in phases with similar characteristics [41].

Chauvet and colleagues reported the first two clinical cases of real-time AR technology in a laparoscopic myomectomy, in which uterine muscle fibers were visualized with diffusion tensor imaging (DTI) after MRI tractography to help surgeons decide whether to proceed with incision [42]. The authors note that AR and DTI fiber tractography are feasible in a uterus with fibroids. They allow for fiber orientation and help the surgeon visualize and decide on the starting point for laparoscopic myomectomy. Attention to fiber orientation may improve scar quality and reduce postoperative architectural disorganization of the uterus [42].

Brebant et al. presented a simplified method of supermicrosurgical lymphovenous anastomosis (LVA) in 30 patients with secondary upper extremity lymphedema [43]. A surgical microscope with an integrated near-infrared illumination system and an AR imaging system was used to evaluate lymphatic supermicrosurgery. The authors note that AR is minimally invasive, highly effective, and has a very low complication rate. The practice of AR guidance is limited by surgical/equipment-related factors and its effectiveness is limited by technical limitations [43].

### 3.2. Improving Education and Training

Of the 31 articles identified, six dealt with education and training. One study was a web-based survey, one study was a randomized controlled trial, one study was a clinical investigation, and three studies were review articles (non-systematic) with insufficiently reported quantitative measures.

In their article, Balla and colleagues investigated the current knowledge and use of AR, VR, and MR technology to improve MIS procedures in surgical training in Italy [44]. A web-based survey was developed. Responses from 217 physicians were analyzed. Participants were surgeons (138, 63.6%) and surgical residents (79, 36.4%). Mean knowledge of the role of VR, AR, and MR in surgery was 4.9 ± 2.4 (range 1–10). Most participants (122, 56.2%) had no prior experience with the proposed technologies. The authors conclude that the level of knowledge and diffusion of these new technologies is still limited in Italy. Further studies are needed to determine the benefits of AR, VR and MR in surgical training [44].

Wild et al. described the iSurgeon system designed for visual guidance in the operating room via telemetry with AR [45]. Novice laparoscopic surgeons (n = 60) were randomized into two groups in a crossover design: Group 1 trained with verbal guidance only and then with additional telestration with AR on the operating room screen, and vice versa for Group 2. Training consisted of basic laparoscopic training followed by a specially designed training course that included a porcine laparoscopic cholecystectomy (LC). The authors suggest that telestration with AR improves training success and safety in MIS [45].

Gholizadeh and colleagues present a literature review on image guidance in liver surgery, with particular emphasis on information on AR techniques [46]. The results show that the use of AR technology can visualize blood vessels and tumor structures in the liver during surgery and enable precise navigation in complicated surgical procedures. AR has been shown to be safe and effective in both minimally invasive and invasive liver surgery. With recent advances and significant efforts by liver surgeons, AR technologies have the potential to dramatically improve hepatobiliary surgery. However, further clinical studies are needed to evaluate AR as a tool to reduce postoperative morbidity and mortality [46].

In their paper, Godzik et al. described how the disruptive technologies of VR and AR are being used in spine surgery and education [47]. According to the authors, initial experiences with VR and AR technologies demonstrate their applicability and ease of implementation. However, further prospective studies in multi-institutional and industry-academic partnerships are needed to clarify the future of VR and AR in spine surgery education and clinical practice [47].

Benčurik and colleagues described the clinical use of near-infrared (NIR) fluorescein angiography, a technique of AR, in the context of anastomotic leak rate (ALR) in rectal resections [48]. Data analysis of patients after MIS for middle and lower rectal adenocarcinoma with total mesorectal excision (TME) using fluorescence angiography (FA) with indocyanine green (ICG) (100 patients) was compared with a historical control group (100 patients). The authors concluded that the introduction of new procedures and the use of new technologies, such as the use of FA in AR mode for rectal resections with TME for cancer, may lead to a reduction in the incidence of anastomotic leakage [48].

In their review, Pratt et al. describe a number of important new imaging technologies, including AR technologies, that are more or less integrated with transoral robotic surgery (TORS) [49]. The authors conclude that image guidance during TORS procedures is an exciting proposition in terms of registration accuracy because the regions of interest (e.g., base of the tongue, or oropharynx) are typically adjacent to and surrounded by rigid anatomy [49].

### 3.3. Building Improved User-Environment Interfaces

Of the 31 articles identified, nine addressed the user-environment interface. Of these, seven were experimental or proof-of-concept approaches and two were review articles with no specified search strategy and insufficiently reported quantitative measures.

In their work, Thabit et al. developed an AR-based suture visualization system and evaluated the accuracy and applicability of AR-based navigation for surgical guidance during minimally invasive spring-assisted craniectomy [50]. The authors concluded that the developed AR system has good accuracy (mean distance 2.4 mm) and can be used during surgery. In addition, the system can help in preplanning minimally invasive craniosynostosis surgery to accurately locate the cranial sutures instead of manually palpating them as in the past [50].

Stewart et al. hypothesized that an AR headset that provides a 3D intracorporeal view while pointed at the surgical field could shorten the time and improve the accuracy of robotic bedside tasks [51]. Bedside assistants (a physician assistant, a medical student, three surgical residents, and two attending surgeons) performed validated tasks in a simulated abdominal cavity with a docked surgical robot. Tasks were performed using a bedside monitor with 2D or 3D vision or an optical head-mounted AR device with 2D or 3D vision. The authors concluded that high-resolution 3D AR vision reduced time and improved accuracy for more complex tasks [51].

In their report, Rush et al. describe institutional experiences with the use and implementation of some of the current AR products in spine surgery [52]. Suggested benefits of AR include reduced distraction, reduced surgical line disruption, the ability to perform more minimally invasive procedures, reduced radiation exposure to the surgical team, and improved pedicle screw accuracy [52].

Previous AR applications for robotic minimally invasive surgery have mainly focused on overlaying preoperative 3D images with patient anatomy. The article by Forte and colleagues presents alternative interactive AR tools for robotic surgery [53]. They designed, built, and evaluated four voice-activated features: Displaying live video from the operating room, displaying two-dimensional preoperative images, measuring 3D resections, and warning of invisible instruments. This low-cost system was developed on a da Vinci Si and can be integrated with surgical robots equipped with a stereo camera and viewer. Eight experienced surgeons performed lymphadenectomies in the dry lab and reported that the features improved the procedure. In particular, they appreciated the ability to access the patient’s medical record on demand, measure distances intraoperatively, and interact with the features using voice commands. The authors concluded that these alternative AR features and interaction methods had a positive impact [53].

In their review, Wendler et al. presented the use of novel molecular imaging as one of the pillars of precision surgery [54]. These devices include technologies ranging from artificial intelligence and computer-aided visualization (software) to innovative molecular imaging modalities and surgical navigation (hardware) [54].

Li and colleagues addressed the current challenges of AR and MR in MIS navigation [55]. The surface of soft tissue is smooth and watery with sparse texture, specular reflection, and frequent deformation. As a result, we often obtain sparse feature points that lead to erroneous results using conventional imaging techniques. The authors present an accurate and robust description and matching method for dense feature points in endoscopic videos. Experimental results show that the novel approach can overcome the influence of specular highlights and robustly describe contours from image sequences of soft tissue surfaces. Compared to state-of-the-art feature point description and matching methods, the presented analysis framework shows the key advantages of robustness and accuracy in matching dense point-to-point images even when severe soft tissue deformation occurs, and the authors expect that this new approach has great potential for 2D/3D reconstruction in endoscopy [55].

Jia et al. pointed out that real-time AR for MIS without additional tracking devices is a valuable but challenging task, especially in dynamic surgical environments [56]. Numerous different movements between target organs are caused by respiration, cardiac motion, or surgical tools, and often need to be captured by a moving, manually positioned endoscope. Therefore, the authors proposed a 6DoF motion tracking method that takes advantage of the latest 2D target tracking methods as well as nonlinear pose optimization and tracking loss recovery in SLAM technologies and can be embedded in such an AR system. The results show that the proposed method is more robust and accurate compared to ORB-SLAM2 in the presence of motion deflection or motion blur; however, heavy smoke is still an important factor that reduces tracking accuracy [56].

Wang et al. showed that tracking is a critical step in achieving accurate AR during MIS [57]. In addition to visual tracking in traditional medical AR, visual tracking attracts much attention because of its generality. If the 3D model of the target organ can be obtained from preoperative images and under the assumption of model rigidity, tracking is transformed into a problem of computing the 6-degree-of-freedom pose of the 3D model. The authors present a robust tracking algorithm in an endoscopic AR system that combines the advantages of regional and dense cues in a unified framework. In addition, an appearance matching method and an occlusion processing method are proposed to effectively handle occlusions. Experiments using a synthetic dataset and a simulated surgical environment demonstrate the effectiveness and robustness of the proposed method. This work presents a novel tracking strategy for medical AR applications [57].

Chen and colleagues point out that previous research on the use of AR technology in monocular surgical MIS scenes has mainly focused on superimposing information without addressing proper spatial calibration, which could lead to mislocalization of annotations and labels, as well as inaccurate depth information and tumor measurements [58]. The authors present a novel intraoperative dense surface reconstruction system capable of providing geometry information from monocular MIS videos for geometry-aware AR applications such as position measurements and depth information. The authors conclude that the new framework is robust and accurate when dealing with challenging situations such as rapid endoscopy camera movements in monocular MIS scenes. Both camera tracking and surface reconstruction based on a sparse point cloud are effective and work in real-time. This demonstrates the potential of the new algorithm for accurate AR localization and depth augmentation with geometric cues and correct surface measurements in MIS with monocular endoscopes [58].

**Table 1:** Categorization of scoping literature search results in relation to the different MIS practice areas.

Citation	Research Topic	Methodology	Outcome Parameter	Findings	Conclusions
			**Navigation**		
Zadeh et al. [28]	AI system for laparoscopic AR-guided uterine surgery	Experimental N = 3800 (images)	Semiquantitative	Segmentation scores: (The higher, the better): 94.6% (training dataset) 84.9% (test dataset) Contour error on training: (The lower, the better): 19.5% (training dataset) 47.3% (test dataset)	System is useful for all surgical steps
Xu et al. [29]	Advantages and disadvantages of various surgical tracking systems	Review Specified search strategy N = 174 (included)	Objective evaluation	Overview of surgical navigation systems, tracking technologies, and preoperative planning procedures	Information loss is a major problem
Butler et al. [30]	In vivo percutaneously inserted pedicle screws with AR guidance	Prospective multicenter clinical trial N = 164 (patients)	Quantitative	Time from registration/percutaneous approach to screw placement: 3.54 min/screw Time per screw placement in first 20 cases: 4.1 min Time per screw placement in last 20 cases: 3.52 min (No difference, *p* = 0.48)	Confirmed efficiency/safety of screw placement with the benefits of AR technology
Zhu et al. [31]	Dual-mode AR-navigated neuroendoscopy for target localization and hematoma removal	Proof-of-concept Experimental N = 24	Quantitative	Root Mean Square Error (RMSE) Between medical images and patients: 0.784 mm Variance: 0.1426 mm Pixel mismatching degrees: <1% in different AR modes Error of distance in catheter implantation experiments: 1.28 mm Variance: 0.43 mm Average error angle: 1.34° Variance 0.45°	High accuracy and feasibility of the system to provide stereo images with depth information fused to the patient
Lecointre et al. [32]	AR-based robotic assistance system for laparoscopic detection of target lymph nodes (TLN) in pelvic lymphadenectomy	Proof-of-concept Animal study N = 2 (pigs)	Quantitative and semiquantitative	CT overlay accuracy: >90% Overflow rates: <6% Significant higher scores: TLN: AR score 3.9 ± 0.32 vs. direct vision; DV, 2.1 ± 0.74 (*p* < 0.001) Ureter: AR score 3.7 ± 0.48 vs. DV 2.5 ± 0.84 (*p* = 0.003) Vessels: AR score 3.4 ± 0.51 vs. DV 1.7 ± 0.67 (*p* < 0.001)	AR approach with rigid registration is a first step in simplifying complex procedures and improving surgical safety
Guo et al. [33]	AR-guided MIS approach for scapula fractures	Retrospective clinical trial N = 21 (patients)	Quantitative	Virtual simulation time: 44.42 ± 15.54 min Time required for pre-operative plate contouring: 16.08 ± 5.09 min AR-guided MIS: Shorter operation time (−28.75 min, *p* = 0.0007) Less blood loss (−81.94 mL, *p* = 0.0052) Similar follow up outcome (*p* > 0.05)	Effective and reliable method for treating scapula fractures
Felix et al. [34]	AR-guided (VisAR) implantation of thoracolumbar pedicle screws	Experimental N = 7 (cadavers)	Quantitative	124 pedicle screws in total Accuracy: 96% (Gertzbein-Robbins grades A and B) Combined angle of error: 2.4° Distance error: 1.9 mm	High-precision, emerging technology for navigating open surgery and MIS techniques with off-the-shelf headset hardware
Yuk et al. [35]	Advances/Applications of AR in spine surgery	Review Specified search strategy N = 41 (included)	Objective evaluation	No randomized controlled trials to date to evaluate accuracy, cost-effectiveness, and patient outcomes. VR training is an effective way to teach traditional/new methods of spine surgery	The use of VR/AR will increase in spine surgery
Chen et al. [36]	In-situ AR navigation system with enhanced arthroscopic information for MIS knee surgery	Experimental N = 2 (knee phantom, swine knee)	Quantitative	Mean targeting error Knee phantom: Traditional 2D arthroscopy navigation: 4.11 ± 0.80 mm AR navigation: 2.01 ± 0.65 mm (Significant difference, *p* < 0.01) In vitro swine knee: Traditional 2D arthroscopy navigation: 5.67 ± 0.97 mm AR navigation: 2.97 ± 0.79 mm (Significant difference, *p* < 0.01)	Suggested AR navigation is helpful in MIS knee surgeries
Benmahdjoub et al. [37]	AR in craniomaxillofacial surgery	Systematic Review Specified search strategy N = 7067 (reviewed) N = 39 (included)	Objective evaluation	Classification of study types, surgery types, equipment used, metrics reported, and benefits	Difficult to aggregate metrics. Difficult to obtain statistical value. Lack of user evaluation studies
Hussain et al. [38]	AR technology in cranial base surgery	Systematic review Specified search strategy N = 210 (reviewed) N = 45 (included)	Objective evaluation	Evaluate the benefits/challenges/solutions of AR systems in cranial base surgery	Growing interest in AR systems that can lead to safer and more cost-effective procedures, but issues need to be addressed
Hussain et al. [39]	Navigation in MIS and its evolution over time	Review Unspecified search strategy N = 54 (included)	Objective evaluation	Overview of the characteristics of navigation in MIS over time and key features for surgical advancement	New developments will further enhance the value of 3D navigation in MIS
Hu et al. [40]	Percutaneous Vertebroplasty (PVP) with the ARCASS AR System	Prospective case-control study N = 18 (patients)	Quantitative	ARCASS group/control group: Less frequency of fluoroscopy (6 vs. 18, *p* < 0.001) Shorter operation time (78 s vs. 205 s, *p* < 0.001) Higher proportion of ‘good’ entry point on lateral views (81.8% vs. 30.0%, *p* = 0.028) and anteroposterior views (72.7% vs. 20.0%, *p* = 0.020)	The ARCASS system provides a more precise bone entry point with less surgical time and unnecessary radiation exposure
Gribaudo et al. [41]	Development of AR-guided robotic surgery	Experimental N = not specified	Objective evaluation	Modular approach to the tracking problem. Segmentation of the entire process into several stages	May be helpful in surgical implementation
Chauvet et al. [42]	AR and magnetic resonance diffusion tensor imaging (DTI) for uterine fiber visualization and tracking	Case series N = 2 (patients)	Clinical evaluation	Localization of myomas Visualization and overlay of uterine muscle fibers	Can help surgeons identify and determine the starting point for laparoscopic myomectomies
Brebant et al. [43]	AR-guided supermicrosurgical lymphovenous anastomosis (LVA)	Clinical trial N = 32 (patients)	PROMs	63 LVAs in total 27 upper extremities 5 lower extremities Mean operation time: 60–150 min Patency was confirmed by intraoperative AR-ICG No postoperative complications	AR-ICG enables a robust validation of LVA
			**Education and training**		
Balla et al. [44]	Knowledge and prevalence of AR in surgical training in Italy	Web-based survey N = 217 (participants)	Quantitative	Participants: University hospital (41%), general hospital (35%), national health system (6%), general surgery (86%), abdominal surgery (72.8%) Knowledge of technology: Mean perceived knowledge (4.9 ± 2.4, out of max. 10), no experience (56.2%), primarily used for training (31.3%), didactic (29%) and intraoperatively (12.4%), Never used before (48.4%) Interest in technology: Should be used for teaching, training, and clinical use (80.3%), significant contribution in training (84.3%) and didactic (71.9%) Limits of technology: Insufficient knowledge (83.9%) and costs (80.6%)	Knowledge and dissemination still limited
Wild et al. [45]	AR-telestration for laparoscopic MIS training	Randomized controlled trial N = 60 (participants) Global Operative Assessment of Laparoscopic Skills (GOALS) Objective Structured assessment of Technical Skills (OSATS) Subjective workload (NASA-TLX questionnaire)	Quantitative	Faster training time (AR vs. verbal guidance) (1163 ± 275 vs. 1658 ± 375 s, *p* < 0.001) Reduced error rates Better laparoscopic cholecystectomy (GOALS 21 ± 5 vs. 18 ± 4, *p* < 0.007 and OSATS 67 ± 11 vs. 61 ± 8, *p* < 0.015) Less complications (13.3% vs. 40%, *p* < 0.020) Reduced subjective workload and stress (33.6 ± 12.0 vs. 30.6 ± 12.9, *p* < 0.022)	AR-telestration improves training success and MIS-safety
Gholizadeh et al. [46]	Overview of MIS and conventional liver surgery based on AR training	Review Specified search strategy N = 135 (review) N = 31 (included)	Quantitative	Inconsistency between algorithms used and claimed registration accuracy (mean 5.38 mm, range 0.93–10.3 mm) Any AR system (manual, semi-automatic, or automatic) requires human input/knowledge. Methods for determining accuracy are inconsistent. Measurements include pixel-based or spatial 3D registration error. Registration accuracy is difficult to determine. Few patients have undergone AR surgery. AR in soft tissue surgery cannot accurately register the virtual model	Further clinical studies are needed to evaluate AR as a tool to reduce postoperative morbidity and mortality
Godzik et al. [47]	VR and AR interfaces in spine surgery and education	Review and case report Unspecified search strategy N = 38 (included)	Objective evaluation	Overview of potential future applications and demonstration of the feasibility of a VR program for neurosurgical spine training using a case study	VR/AR is easy to implement. Further prospective studies through multi-institutional and industry-academic partnerships are needed to solidify the future of VR/AR in spine surgery education and clinical practice
Benčurik et al. [48]	New procedures and technologies for total mesorectal excision (TME)	Clinical trial N = 200 (patients)	Semiquantitative	In fifteen patients (15%), resection was postponed due to inadequate perfusion detected by AR. The incidence of anastomotic leakage was lower in the group with AR than in the group without AR (9% vs. 19%, *p* = 0.042)	The use of AR in rectal resections with TME for cancer may lead to a reduction in the incidence of anastomotic leakage
Pratt et al. [49]	Image guidance and AR in transsoral robotic surgery	Literature Overview and recent appraisals N = 10 (included)	Objective evaluation	Preoperative imaging guidance Intraoperative fluorescence imaging. Deformable registration using CBCT imaging. Image guided cochlear implantation	Ability to expand the surgical field with navigational cues and visualization of important anatomical structures
			**User-environment interface**		
Thabit et al. [50]	AR with electromagnetic tracking system for MIS craniosynostosis	Experimental N = 120 (sutures on two skull phantoms) System Usability Scale (SUS)	Quantitative	Distance of the marked sutures from planning reference: 2.4 ± 1.2 mm Time per suture: 13 ± 5 s SUS value: 73	Good accuracy Helpful in pre-planning MIS craniosynostosis surgery
Stewart et al. [51]	AR system for bedside surgical assistance	Proof-of-concept N = unspecified Different bedside tasks with da Vinci Xi surgical system on mock abdominal cavity	Semiquantitative	Improved times for ring path task with better resolution: lower resolution 23 ± 11 s vs. higher resolution 14 ± 4 s (*p* = 0.002)	High-resolution AR reduces time and improves accuracy during more complex laparoscopic procedures
Rush III et al. [52]	Advantages/disadvantages of AR in spine surgery	Review Unspecified search strategy N = 20 (included)	Objective evaluation	Different AR systems: Augmedics and Holosurgical ARAI navigation system	Accurate anatomical information with minimal to no radiation exposure
Forte et al. [53]	Voice-activated system for displaying live video on da Vinci Si surgical robot	Experimental N = 8 (surgeons) Phantom model Utility and usability questionnaire Four voice-controlled AR functions: Viewing live video Viewing 2D pre-op images Measuring 3D distances Warning about out-of-view instruments	Quantitative and semiquantitative	Average time for surgeons to become familiar with the technology: 8.47 min Accuracy of voice commands: 100% Voice command sensitivity: 89.8%	Support for further exploration
Wendler et al. [54]	Evaluate new technologies at various stages of the surgical workflow	Review Unspecified search strategy N = 226 (included)	Objective evaluation	Artificial intelligence. Computational visualization. Innovative molecular imaging modalities. Surgical navigation	Integrating molecular imaging could be the key to a new level of precision surgery
Li et al. [55]	Dense feature point description and matching method in endoscopic video	Experimental N = 3 (video segments)	Quantitative	True Positive Matching Result (TPM): 142.33 False Positive Matching Results (FPM): 10	New approach has great potential for 2D/3D reconstruction in endoscopy
Jia et al. [56]	6DoF method to improve motion tracking in AR systems	Experimental N = unspecified Ex-vivo tissue phantoms (kidney) and clinical datasets Root Mean Squared Error (RMSE)	Quantitative	RMSE: 2.31 mm (without disctraction) RMSE: 3.43 mm (middle-level distraction) RMSE: 3.56 mm (high-level distraction)	Robust and long-term tracking in highly dynamic operating environments
Wang et al. [57]	Robust tracking algorithm in an endoscopic AR system	Experimental N = unspecified Experiments with synthetic and simulation datasets	Quantitative	Average Contour Distance: 1.2398 pixels Frame Rates: 38.46 fps	The effectiveness and robustness of the method represents a novel tracking strategy for medical AR
Chen et al. [58]	Robotic algorithm (SLAM) in monocular surgical MIS scenes for reliable endoscopic camera tracking	Experimental Simulated laparoscopic scene image sequences and clinical data (N = 877) Root Mean Square Distance (RMSD)	Quantitative	RMSD: 2.54 mm Other monocular MIS scene reconstruction method (RMSD: 7.21 mm) State-of-the-art stereo reconstruction method (RMSD: 2.04/2.57 mm)	High accuracy of the developed algorithm

## 4. Discussion

AR technology is gaining increasing interest in the development of minimally invasive surgery today [19]. This is reflected in the number of research articles published in recent years from a variety of medical disciplines (Table 1). Although many studies have been published on this topic, some of them differ fundamentally in their research questions, data collection methods, and design, making it impossible to compare them in meta-analyses. In addition, most studies to date have been experimental and few have provided significant clinical benefits to patient care.

Most of the developments have been made in spine surgery/orthopedic surgery [30,33,34,35,36,40] and for endoscopic/laparoscopic procedures [28,31,32,33], which have not yet fully exploited the potential of this new technology. AR could help identify and visualize critical anatomical structures such as blood vessels and nerves to reduce the risk of injury or to guide the proper placement of surgical instruments [30,31].

To answer the question of how AR technology can help improve MIS procedures, we have identified three main areas of application. Most research today is focused on intraoperative AR-guided navigation. AR technology is also being used to improve education and surgical training and to develop and improve new user-environment interfaces.

While the studies discussed here indicate some potential of AR technology in the context of improving MIS navigation solutions [28,29,30,31,32,33,34,35,36,37,38,39,40,41,42,43], current AR-guided navigation systems have not shown improved precision compared to conventional navigation methods, albeit shown improvements in ergonomics and visualization have been reported [38]. However, what has been clinically demonstrated to date is evidence of shorter operative times and less blood loss, suggesting a gentler surgical approach [33,40]. Most of the AR-guided navigation studies presented here are experimental development approaches, some of which have shown promising results [28,31,32,34,36,41]. However, clinical trials with adequately sized numbers of patients or even systematic reviews demonstrating added value for patient care are still rare [30,33,37,38].

Although AR is predicted to revolutionize surgery, there are several challenges that need to be addressed to ensure widespread adoption [55,56,57,58]. A major issue, especially in soft tissue navigation, is the accuracy and reliability of the AR system, particularly in registering patient data and matching virtual and real views [55,56]. These are prone to errors, especially during intraoperative repositioning. It is moreover important for future research to identify factors for evaluating AR accuracy. For this purpose, Root Mean Square Distance (RMSD) or Root Mean Square Error (RMSE) has been proposed by some authors [31,56,58]. In addition, real-time processing, and visualization of a large amount of data for AR navigation is problematic because it is computationally intense and requires advanced hardware and software infrastructure [30,31,32,33,34,35,36,37,38,39,40,45,50]. Nevertheless, some promising developments in robotics, visualization, positioning, haptics, artificial intelligence, and computer vision have been presented that may help to further advance AR technology for clinical use [55,56,57,58].

Several studies have addressed the benefits of AR technology and its ability to enhance surgical education and training by providing students and professionals with an immersive, interactive, and engaging learning experience [20,44,46,47,51]. However, awareness of this technology among clinically active surgeons remains low [44]. Without requiring expensive equipment or putting patients at risk, trainees can practice and refine surgical procedures using virtual models of patient anatomy [45]. They receive real-time feedback on their performance, allowing them to refine their technique and improve patient outcomes in vivo. In addition, AR can be used to train surgeons in new or rare surgical procedures that are difficult to perform in the real world [46]. The benefits of virtual surgical training could, therefore, indeed impact MIS approaches. However, this influence has yet to be demonstrated via standardized studies with a large number of participants.

The interface between users and different surgical environments can be improved by using AR technology to provide relevant and contextual information in real-time [19,21,22,23,51,53]. Surgeons can visualize patient data and imaging scans in 3D, allowing for more accurate and personalized surgical planning [23]. It can enable remote collaboration and consultation between surgeons, facilitating knowledge sharing and improving patient outcomes [51]. However, to date, most studies on the topic of user interface improvement have been experimental or proof-of-concept approaches. So far, there is no evidence of added value for patient care.

## 5. Conclusions

AR technology is currently being investigated for MIS approaches in various medical disciplines. Most studies to date have been limited to experimental developmental approaches, while the number of clinical trials and systematic reviews is low. Most articles on AR-guided navigation are limited to endoscopic/laparoscopic MIS approaches. Intraoperative tracking is still considered cumbersome and error prone. In addition, processing large amounts of data in real-time is computationally intensive and requires improved hardware and software solutions. However, AR technology has been shown to improve ergonomics and visualization, as well as reduce operation time and blood loss. AR can also be used to improve education and surgical training and to create and improve new user interfaces that may indirectly improve MIS procedures. However, to date, controlled studies with large case numbers and standardized outcome parameters and reporting are lacking to confirm the added value for clinical use.

## Data Availability

Data are available from the corresponding author.
